# PTSD symptoms as a consequence of breast cancer diagnosis: clinical implications

**DOI:** 10.1186/2193-1801-3-392

**Published:** 2014-07-29

**Authors:** Paola Arnaboldi, Claudio Lucchiari, Luigi Santoro, Claudia Sangalli, Alberto Luini, Gabriella Pravettoni

**Affiliations:** Applied Research Unit for Cognitive and Psychological Science, European Institute of Oncology, Via Ripamonti, 435, 20141 Milan, Italy; Department of Epidemiology and Biostatistics, European Institute of Oncology, Via Ripamonti, 435, 20141 Milan, Italy; Division of Senology, Data Management, European Institute of Oncology, Via Ripamonti, 435, 20141 Milan, Italy; Department of Health Sciences, University of Milan, Via A. di Rudinì, 8, 20142 Milano, Italy

**Keywords:** Newly diagnosed breast cancer patients, Patient healthcare professional relationship, PTSD symptoms, Patient management, Cancer familial history

## Abstract

It is a well-established multidisciplinary practice at the European Institute of Oncology, that nurses and physicians often report their difficulties to clinical psychologists regarding adherence to hospital scheduling and procedures, when faced with women who, having been diagnosed with cancer, may be too overwhelmed to understand medical advice. We thus undertook an observational-prospective-cohort study, to investigate the prevalence and variation of PTSD symptomatology in women awaiting a mastectomy at a mean of 30 days after diagnosis and up to 2 years after discharge from hospital. The presence of any correlations between PTSD symptoms and medical and psycho-social variables was also investigated. Between March 2011 and June 2012, 150 women entered the study and were evaluated at four points in time: pre-hospital admission, admission for surgery, hospital discharge and two years later. The prevalence of distress at pre-hospital admission was 20% for intrusion symptoms, 19.1% for avoidance symptoms and 70.7% for state anxiety. Intrusion was negatively correlated with time from diagnosis independently of tumor dimensions, i.e. independently of the perceived seriousness of the illness. Even though at two-year follow up the prevalence of intrusion and avoidance is similar to that in the general population, patients with high levels of intrusion and avoidance at pre-hospital admission will maintain these levels, showing difficulties in adjusting to illness even two years later. As for psycho-social factors, the presence of a positive cancer family and relational history is associated with high levels of distress, in particular with intrusive thinking. Proper interventions aimed at the management of these issues and at their implications in clinical practice is clearly warranted.

## Background

Nowadays, the psychological consequences of a cancer diagnosis are well-recognized worldwide. A consensus is yet to be reached upon the most suitable approach to handle such consequences in order to deliver high quality, multidisciplinary care.

Regarding the epidemiological aspects, in one study carried out at a Comprehensive Cancer Center, involving nearly 4500 patients aged 19 and older, the prevalence of significant psychological distress ranged from 29 percent to 43 percent for patients with the 14 most common types of cancer (Zabora et al. ( 2001). Other studies have reported high rates of psychological symptoms, meeting criteria for such clinical diagnoses as depression, adjustment disorders and anxiety (Carlsen et al. 2005; Hegel et al. 2006). From one recent meta-analysis by Mitchell et al. ( 2011), it emerged that 20-25% of cancer patients have clinically significant levels of depression, anxiety and adjustment disorders that interfere with treatment outcomes.

In 1994, the American Psychiatric Association (APA) recognized diagnosis with a life-threatening illness, such as cancer, as a traumatic stressor that could precipitate another psychological syndrome: the post-traumatic stress disorder (PTSD) (American Psychiatric Association 1994). Research has shown that such a syndrome appeared to be a better model for conceptualizing distress in patients with cancer than did a generalized “distress” model (Rustad et al. 2012).

The Diagnostic and Statistic Manual of Mental Disorders - V edition (American Psychiatric Association 2013) includes PTSD in a new chapter on trauma and stress-related disorders: PTSD, regardless of its trigger event, is characterized by clinically significant distress or impairment in the individual’s social interactions, ability to work, or in other key areas of functioning. Symptoms that accompany PTSD now fall into four distinct diagnostic clusters, instead of three (American Psychiatric Association 1994): re-experiencing, avoidance, negative cognitions and mood, and arousal.

Re-experiencing covers spontaneous memories of the traumatic event, flashbacks or other intense memories, or prolonged psychological distress. Avoidance refers to distressing memories, thoughts, feelings or external reminders of the event. Negative cognitions and mood represent feelings ranging from a persistent and distorted sense of blame of self or others, estrangement from others or markedly diminished interest in activities, to an inability to remember key aspects of the event. Finally, arousal is marked by aggressive, reckless or self-destructive behavior, sleep disturbances, hyper-vigilance or related problems.

It emerges from the literature that up to 19% of adults receiving a cancer diagnosis present a PTSD-like syndrome when assessed using the symptom cluster method (Kwekkeboom and Seng 2002; Elklit 2002; Kwakkenbos et al. 2014). According to the literature, predictors of PTSD symptomatology in cancer patients include psychological disturbances prior to cancer diagnosis, elevated psychological distress subsequent to diagnosis, younger age, female gender, lower socioeconomic status, poor social functioning and support, and avoidant coping style (Kangas et al. 2005; Mehnert and Koch 2007). Chronic PTSD is associated with higher risk for physical morbidity (Buckley et al. 2004) and can also lead to nonadherence with medical treatment, impacting local and distant recurrences (Ma et al. 2008). In this regard, Jacobsen and colleagues state that the presence of PTSD symptoms can explain “non-compliant” patients’ behavior (Jacobsen et al. 1998). Early detection of PTSD symptoms can therefore help both psychological health and medical prognosis. In particular, avoidant reactions seem to be more associated with poorer physical health while intrusive reactions seem to be related to poorer psychological health (Lutgendorf et al. 2012). Avoidant individuals are less likely to seek support, to complain about physical symptoms and, thus, to be compliant with the process of care (Ciechanowski et al. 2002). Detecting the presence of such symptoms as early as possible in the patient’s clinical pathway can have important advantages on clinical outcomes and allows one to understand where psychological intervention is most needed. It could be that the accumulation of the perceived traumatic events is related to the prevalence of PTSD (O’Connor et al. 2011). In the study by O’Connor, breast cancer patients were evaluated for PTSD symptoms at 3 and 15 months after surgery but the time of diagnosis is undoubtedly also a very sensitive moment.

It is interesting in this regard, that the study by Mundy and colleagues (Mundy and Baum 2004) found no current PTSD in the sample of breast cancer survivors, yet 35% of the sample was diagnosed with lifetime PTSD. This could be due to the fact that, at the time of the study, participants were at least 100 days post-treatment.

Luecken et al. ( 2004) assessed the prevalence of PTSD within six months after diagnosis in a sample of 71 women. Only 3% of the sample met the criteria for PTSD due to breast cancer.

To the best of our knowledge, there is no evidence from the literature of studies in which psychological distress and PTSD symptoms have been assessed within the first month from the very moment of diagnosis. This represents a fruitful area of investigation from a health care perspective.

At the European Institute of Oncology (IEO), newly-diagnosed breast cancer patients can await surgery for 1 to 4 weeks from the time of diagnosis. This is a very difficult period to cope with and it is characterized by fears, anguish and anxiety.

Nurses and physicians at the IEO are regularly faced with newly-diagnosed women who may be too confused and overwhelmed to understand medical advice, rendering them indecisive at a time when their participation in the process of care is clearly warranted. In accordance with well-established IEO practice, the healthcare professional will often report their difficulties to clinical psychologists regarding the need to adhere to hospital scheduling and procedures. Furthermore, healthcare professionals commonly tend to evaluate the impact that cancer has on a patient's psychological distress as being determined by the severity of the cancer. In this regard, tumor dimension at diagnosis could be considered determinant.

We hypothesized that the presence of PTSD symptoms at the very time of breast cancer diagnosis could be responsible for difficult future adjustment throughout the process of care and also for poor psychological outcomes.

In this context, our primary purposes were:

- To evaluate the incidence of PTSD symptoms a mean of 30 days after diagnosis
- To evaluate how PTSD symptoms change over time, from diagnosis to hospital discharge after mastectomy until two-year follow up

Our secondary outcomes were:

- To evaluate correlations between sample socio-demographic characteristics
- To evaluate correlations between perceived emotional support and psychological distress over time

## Results

### Sample

Between March 2011 and June 2012, 150 women entered the study. Of these, 83 completed the 2 year follow-up while 67 did not complete follow-up. In both groups there were 26 patients who did not complete the questionnaire at the T2 time-point.

Failure to complete T2 was mostly due to organizational aspects (such as early hospital discharge, other medical procedures).

The median age of the sample was 47 years [30–81] (see Table 1). As regards civil status, 30% were single, divorced or widowed, while 70% were married or cohabitant. Twenty-nine percent had a lower secondary school education while 71% an upper secondary school education. A total of 26% came from the Lombardy region while 74% from outside Lombardy. Altogether, 83.3% of the sample had a relative or a significant other who had suffered from cancer. Regarding tumor dimension, 44% (n = 66) had T2, 32% (n = 48) had T1, 1.3 (n = 2) had T0, while for 22.7% (n = 34) the data was missing. When women were asked if they had suffered or were suffering from a psychological disturbance, 20% (n = 30) answered affirmatively (see Table 2).

**Table 1:** **Socio-demographic characteristics**

Variables	N	%
Age (years)		
<40	24	16.0
40-49	68	45.3
50-59	33	22.0
60-69	15	10.0
≥70	10	6.7
Age (continuous)		
**Mean (sd)**		49.2 ± 10.9
**Median (min – max)**		47 (30–81)
**Civil status**		
Unmarried	23	15.3
Cohabitant	9	6.0
Married	96	64.0
Divorced	15	10.0
Widow	7	4.7
**Children**		
Yes	111	74.0
No	39	26.0
**Education**		
Elementary	10	6.7
Secondary	33	22.0
High	74	49.3
Degree	33	22.0
**Geographic area/1**		
Lombardy/out of Lombardy	26.0	74.0

**Table 2:** **Clinical issues**

Variables	N	%
Tumour size as referred by the patient		
T0	2	1.3
T1	48	32.0
T2	66	44.0
Unknown	34	22.7
**Psychological treatments**		
Yes	12	8.0
No	138	92.0
**Family history for tumour**		
Yes	125	83.3
No	25	16.7

At baseline, the median time elapsed from diagnosis was 30 days (n = 150) while the median time that women had to wait from pre-hospital admission to operation (n = 114) was 14 days.

### Emerging issues

The prevalence of distress at pre-hospital admission, approximately 30 days after diagnosis, was 20% for intrusion symptoms, 19.1% for avoidance symptoms and 70.7% for state anxiety (Table 3).

**Table 3:** **Intrusion, avoidance and anxiety over time, as continuous variables (all available data)**

Variables	Time				p-value ^*^
	Before admission	At admission	At discharge	2-year follow-up	
IES questionnaire	N = 150	N = 114	N = 98	N = 83	
**Intrusion**					
Mean (sd)	22.1 (5.1)	20.8 (5.4)	20.5 (6.4)	19.4 (4.4)	
Median (min-max)	22 (10–35)	21.5 (9–35)	20 (9–39)	20 (9–31)	0.001
**Avoidance**					
Mean (sd)	13.8 (4.3)	13.5 (4.9)	12.7 (5.0)	11.4 (4.1)	
Median (min-max)	14 (6–24)	13 (6–26)	12 (6–24)	11 (6–23)	0.0008
STAI questionnaire	N = 150	N = 110	N = 97	N = 82	
**Anxiety**					
Mean (sd)	47.0 (12.0)	48.5 (12.0)	41.4 (11.1)	43.1 (12.0)	
Median (min-max)	45.5 (20–77)	47.5 (22–77)	41.0 (21–76)	42.0 (22–77)	<0.0001

^*^Kruskal-Wallis test.

Anxiety levels and PTSD symptoms varied over time, from pre-hospital admission to 2-year follow up: 70% of patients had clinically significant anxiety at the very time of diagnosis, 76.4% at the moment of pre-hospital admission, and 54.9% at the 2-year follow-up. In particular, a statistical association emerged between avoidance scores at baseline and avoidance scores at two-year follow up.

Among the 83 patients with both pre-hospital admission and 2-year follow-up data, the number of patients with clinically significant state anxiety (>39) significantly reduces over time (p-value =0.002; Table 4).

**Table 4:** **Intrusion, avoidance and anxiety: “2-year follow-up” vs. “before admission” (pre and post data) continuous variables**

	Before admission (A)	2-year follow-up (B)	Difference (A) – (B)	p-value#
IES questionnaire	N = 83	N = 83	N = 83	
	n (%)	n (%)	n (%)	
**Intrusion**				
Mean (sd)	22.3 (4.7)	19.4 (4.4)	2.9 (5.2)	
Median (min-max)	23 (11–33)	20 (9–31)	3 (-12, 16)	<0.0001
**Avoidance**				
Mean (sd)	13.3 (4.0)	11.4 (4.1)	1.9 (4.1)	
Median (min-max)	13 (6–23)	11 (6–23)	2 (-9, 12)	<0.0001
STAI questionnaire	N = 82	N = 82	N = 82	
	n (%)	n (%)	n (%)	
**Anxiety**				
Mean (sd)	47.2 (11.7)	43.1 (12.0)	4.0 (11.4)	
Median (min-max)	45.5 (20–73)	42.0 (22–77)	3.0 (-30, 36)	0.002

Continuous variables.

#t test.

The number of patients with clinically significant intrusion (> = 27) showed a similar statistically significant reduction (p = 0.01) from 20% (17/83) to 7.2% (6/83), while the number of patients with avoidance reduction (from > =18 to <18) did not counterbalance those who showed avoidance worsening (from <18 to > =18 over time), therefore providing a not statistically significant result (p = 0.13, Table 5).

**Table 5:** **Intrusion, avoidance and anxiety: “2-year follow-up” vs. “before admission” (pre and post data)**

	*2*-*year follow*-*up*		p-value~
**Intrusion (N = 83)**			
***Before admission***	<27	> = 27	
<27	62	4	
			0.01
> = 27	15	2	
**Avoidance (N = 83)**			
***Before admission***	<18	> = 18	
<18	68	3	
			0.13
> = 18	8	4	
**Anxiety (N = 82)**			
***Before admission***	<=39	>39	
<=39	13	7	
			0.002
>39	24	38	

Categorical variables

~McNemar test.

However the mean difference of avoidance (+1.9) from pre-hospital admission (13.3) to 2-year follow up (11.4) resulted statistically significant different from zero (p < 0.0001).

Intrusion is negatively correlated with time from diagnosis irrespective of tumor dimensions (p = 0.84) and number of children (p = 0.22). As to psycho-social factors, the presence of a family history seems to be associated (p = 0.07) with higher levels of intrusive thinking over time (+3.4), while just a negligible change is observed in the subgroup of patients with no familiar history (+0.6) (Table 5).

Conversely, tumor dimension, number of children and family history, does not affect avoidance and anxiety change over time (Table 6).

**Table 6:** **Intrusion, avoidance and anxiety: difference “2-year follow-up” vs. “before admission”**

	IES questionnaire (N = 83)					STAI questionnaire (N = 82)		
	N	Intrusion	p-value#	Avoidance	p-value#	N	Anxiety	p-value#
**Children [mean, (sd)]**								
Yes	65	3.3 (5.4)		1.7 (4.1)		65	4.3 (11.6)	
No	18	1.6 (4.6)	0.22	2.4 (4.0)	0.53	17	3.1 (11.1)	0.69
**Family history [mean, (sd)]**								
Yes	69	3.4 (5.0)		2.0 (4.3)		68	4.7 (10.8)	
No	14	0.6 (6.0)	0.07	1.3 (2.9)	0.54	14	0.8 (14.0)	0.25
**T [mean, (sd)]**								
T0-T1	30	3.2 (5.7)		1.9 (4.7)		29	2.6 (13.1)	
T2	32	3.4 (5.0)	0.84*	1.4 (4.0)	0.66*	32	4.6 (10.2)	0.50*
Unknown	21	1.9 (4.9)		2.8 (3.2)		21	5.2 (11.1)	

Subgroup Analysis

^#^t test.

^*^comparing T0-T1 vs T2.

One can say that cancer diagnosis was responsible for causing psychological distress in patients independently of medical factors such as tumor dimension.

It is important to note that taking into consideration the relationship between the dropout behavior and avoidant symptoms, patients who completed all four assessment sessions had a lower probability of presenting clinically relevant avoidant scores (see Table 7).

**Table 7:** **Drop-out “behavior” over time according to AVOIDANCE value at baseline (categorical)**

Variables	Drop-out “behavior” over time				p-value
	No 2-year follow-up		2-year follow-up		
	Dropout before discharge	NO Dropout before discharge	Dropout before discharge	NO Dropout before discharge	
	N = 26	N = 41	N = 26	N = 57	
	n (%)	n (%)	n (%)	n (%)	
**Avoidance at baseline**					
> = 18	6 (23.1)	11 (26.8)	8 (30.8)	4 (7.2)	0.02^*^

^*^chi-square test.

A total of 95% of patients judged the socio-familiar support received at the time of pre-hospital admission as optimal or nearly optimal. We did not find any correlations between this value and psychological distress.

## Discussion

Results from this study are consistent with other published results that show an incidence of PTSD symptoms of around 20% in newly-diagnosed cancer patients (Mundy and Baum 2004; Mehnert and Koch 2007; Mitchell et al. 2011; O’Connor et al. 2011; Rustad et al. 2012), which is very far from the prevalence rate in the general population (Pietrzak et al. 2011).

Even if psychological distress tends to diminish over time, patients with high levels of intrusion and avoidance at diagnosis tend to maintain those levels even at two years after diagnosis. This could cause difficulties in the process of adjustment to illness thus warranting closer attention in a preventive perspective, i.e. at the very beginning of the process of care.

Contrary to the evidence presented in the literature (Kangas et al. 2005 Mehnert and Koch 2007), among psychosocial factors detected, only a relational and/or family history of cancer is associated with distress: neither perceived social support, nor a supportive family are inversely correlated. It seems that patient’s memory can influence her ability to cope with cancer: memories and recollections of cancer experiences of meaningful others seem to overload the patients cognitive and emotional field in an intrusive manner. Evidence of an absence of association between levels of distress experienced and the perceived illness seriousness as described by the TNM tumor classification also points in this direction. In other studies (Zabora et al. 2001; Palyo and Gayle 2006) it emerges that PTSD symptoms are correlated with pain perception and levels of fatigue underlining the importance of the cognitive dimension (E.g. “which resources may I use?”) also in modulating emotional functioning.

## Conclusions

These results have important clinical implications: physicians and surgeons should be made aware of the high prevalence of post-traumatic stress symptoms in their newly-diagnosed breast cancer patients and they, therefore, should be helped in adapting their communication skills to these issues thorough continuing medical education (CME) programs: CME programs could be viewed as offering systematic assistance in identifying patients emerging needs. For example, the Memorial Sloan Kettering Cancer Center runs an advanced communication training program integrated into the institution’s regular practices and continuing medical education (CME) (Bylund et al. 2011).

Our results underline the importance of a standardized measurement of psychological distress being incorporated into the hospital routine with specific attention paid to PTSD symptoms. When a patient shows shock, confusion, carelessness, says she is unable to decide and seems not to remember what physician has just told her, this could be because she is suffering from PTSD symptomatology and detailed attention is thus warranted.

Since 74% of our patients come from outside the Lombardy region, at a considerable physical distance from IEO, programs of care should be created to effectively address psychological issues, for example in the form of telephone or computer-based support delivery. The telephone-based support or information delivery has proved to be beneficial with either patients and relatives in different illness phases (Arnaboldi et al. 2010).

Our study has limits, principally due to the sample size. Furthermore, the dropout rates do not allow us to readily generalize from the results. Nonetheless, our data strongly suggest the need to thoroughly investigate the impact of PTSD symptoms on clinical outcomes in early-diagnosed cancer patients.

In conclusion, we would like to emphasize the importance of a patient-centered approach, which fully takes into account psycho-social and cognitive issues (Lucchiari et al. 2010) as being the most suitable way to improve physicians’ abilities to respond to patients clinical needs.

## Methods

We proposed participation in a study to newly-diagnosed breast cancer patients awaiting mastectomy. It was explained to them that its aim was to monitor their emotional state and their psychological reactions to diagnosis in order to promote better care strategies.

PTSD intrusion and avoidance symptoms and state anxiety were measured by means of the Impact of Event Scale (IES) (Horowitz et al. 1979) and of the State–Trait Anxiety Inventory (STAI) (Spielberger 1989) at four points in time: pre-hospital admission T0 (a mean time of 33 days after diagnosis), surgical admission (T1), discharge from hospital (T2) and two years later (T3).

Nurses receiving patients in outpatient appointments with the surgeon, who had communicated to them their cancer diagnosis and the indication for a mastectomy procedure, asked patients if they were interested in meeting a research psychologist that would present them the study and ask for their written informed consent.

In the case of the patient’s acceptance, the nurse asked for the patients name and surname and for a telephone number on which the research psychologist could contact them. Psychologists contacted the patients and arranged an interview for the scheduled day of pre-hospital admission.

To enter the study, patients had to be aged 18, at their first cancer diagnosis, having received an indication for a mastectomy procedure and be Italian mother tongue. Women with a medical history of major psychological disorders were excluded as were women with an indication for pre-surgery neoadjuvant treatment. The study was approved by the IEO scientific committee and to guarantee anonymity and secrecy, once enrolled, women were assigned with a progressive number.

At pre-hospital admission (T0), which represented our baseline, women were asked to provide socio-demographic information and also to report their perceived emotional support in relation to their cancer experience by means of a 0–10 visual-analogue scale, the presence of significant others (not only relatives) who have had a cancer diagnosis and the presence of previous or current emotional disorders.

Women were also asked to recall the day they received their cancer diagnosis and the tumor dimension according to the TNM classification of breast cancer, as described in their medical chart. Secondly, they were asked to complete the two questionnaires: IES and STAI. The same questionnaires were then administered to women when they entered hospital for surgery and on the day of discharge.

Psychologists and nurses coordinated themselves in organizing, together with patients, appointments to complete the questionnaires on the day of admission and discharge, so as to fit in with the scheduling requirements for medications, check-ups, post-surgery physiotherapy etc.

Approximately two years after hospital discharge, a psychologist contacted by telephone, all women who had initially agreed to participate in the study and conducted a telephone interview with those available. At that time, PTSD symptoms and state anxiety were again assessed, together with perceived emotional support. The psychologist tried to contact each of the enrolled women three times to propose this two-year follow-up telephone interview. After the fourth failed contact, the patient was considered non-respondent.

### Statistical analysis

Descriptive statistics were used to characterize the sample with regard to socio-demographic, tumor and clinical characteristics. Categorical data were expressed as numbers and percentages, while continuous data were presented as mean and standard deviation, median and range.

Pearson’s chi-square for categorical variables and the Kruskal-Wallis test were used when evaluating avoidance, intrusion and anxiety over the four time-points, as both categorical and continuous variables. Differences at 2-year vs baseline were evaluated by the t-test for paired data when the variables were measured on continuous scale, and by the McNemar test when a cut-off was used to dichotomize the variable.

All the analyses were performed with SAS statistical software (SAS Institute, Cary, NC), Version 9.02.
